# A Case of Tip Appendicitis: The Role of CT Scan

**DOI:** 10.7759/cureus.84244

**Published:** 2025-05-16

**Authors:** Pokhraj P Suthar, Pravalya Chaparala, Sumeet Virmani

**Affiliations:** 1 Department of Diagnostic Radiology and Nuclear Medicine, Rush University Medical Center, Chicago, USA

**Keywords:** acute abdominal pain, appendicitis, computed tomography, laparoscopic appendectomy, laproscopy, variants of appendicitis

## Abstract

Acute appendicitis is a common cause of abdominal pain and a leading indication for surgery, especially in younger patients. While its diagnosis is often straightforward, rarer forms like acute tip appendicitis can be more difficult to detect. The growing use of CT imaging has made it easier to identify these unusual cases. A 32-year-old female patient presented with sharp, constant abdominal pain localized to the right lower quadrant and suprapubic region, with an intensity of 7/10 that worsened with oral intake. Physical examination revealed minimal tenderness in the lower quadrants, along with right adnexal tenderness, and lab results, including a negative urine pregnancy test, were unremarkable. CT imaging showed mild dilation of the appendiceal tip and subtle periappendiceal fat stranding, suggesting early tip appendicitis, while the rest of the appendix appeared normal. The patient underwent laparoscopic appendectomy, which confirmed inflammatory changes at the appendiceal tip, thus diagnosing acute tip appendicitis. This case underscores the importance of considering tip appendicitis in the differential diagnosis of right lower quadrant pain, particularly with the increasing use of CT imaging. Despite its rarity, tip appendicitis should be recognized as a potential cause with similar clinical risks as more common forms of appendicitis, including significant morbidity if not treated promptly.

## Introduction

Acute appendicitis remains one of the most frequent causes of acute abdominal pain requiring surgical intervention, particularly in adolescents and young adults. It is estimated that approximately 7% of the population will experience appendicitis at some point in their lives, with peak incidence occurring in the second and third decades [1,2]. The classic presentation includes right lower quadrant pain, nausea, vomiting, anorexia, and localized tenderness, often accompanied by leukocytosis and elevated inflammatory markers [2]. These clinical features typically guide diagnosis and expedite surgical management.

However, atypical and localized variants of appendicitis, such as acute tip appendicitis, present unique diagnostic challenges. In these cases, inflammation is confined to the distal portion (tip) of the vermiform appendix, while the proximal segments may appear normal on both clinical examination and imaging. Such subtle and localized inflammation may lead to diagnostic uncertainty or misdiagnosis, particularly when classic symptoms are absent or minimal [3].

With the increasing availability and use of high-resolution computed tomography (CT), especially in emergency settings, the detection of these atypical cases has improved significantly. CT imaging has been shown to possess high sensitivity and specificity for appendicitis, with some studies citing diagnostic accuracy rates above 90% [4,5]. In a study by Mazeh et al., involving 18 patients with CT findings suggestive of tip appendicitis, only 39% were ultimately confirmed to have appendicitis upon clinical and/or surgical evaluation [4]. Importantly, CT allows for detailed visualization of the appendix, including the tip, which may not be well visualized on ultrasound or detected via physical examination alone [6].

Despite these advances, acute tip appendicitis remains under-recognized due to its relative rarity and less dramatic clinical course. As a result, diagnosis often relies heavily on imaging, and awareness among clinicians remains crucial to ensure timely intervention. Failure to diagnose appendicitis promptly can lead to complications [5].

This report presents a rare instance of acute tip appendicitis in a 32-year-old female who presented with persistent lower abdominal pain in the absence of systemic symptoms such as fever or leukocytosis. It highlights the diagnostic utility of CT imaging in identifying localized appendiceal inflammation and the importance of maintaining a broad differential diagnosis in patients with nonspecific abdominal pain [6,7].

This case was earlier accepted for the Daily Cases program in the American Roentgen Ray Society (ARRS) 2025 Roentgen’s Key Case Challenge and presented at the ARRS 2025 conference in San Diego

## Case presentation

A 32-year-old female patient presented to the emergency department with abdominal pain. She described the pain as constant and sharp, located in the right lower quadrant to the suprapubic region, with an intensity of 7/10. The pain worsened with oral intake and was not relieved with the use of paracetamol. The patient denied any history of nausea, vomiting, bloody stools, fever, urinary symptoms, or vaginal discharge. She was sexually active, monogamous, and used barrier contraceptives. There was no significant history of tobacco, alcohol, or recreational drug use.

On physical examination, the patient exhibited minimal tenderness to palpation over bilateral lower quadrants without rebound, rigidity, or guarding. There was associated right adnexal tenderness. Otherwise, the rest of the physical examination was within normal limits. The urinary pregnancy test was negative, and the ultrasound of the uterus and bilateral ovaries was unremarkable. Vital signs revealed blood pressure 106/51 mmHg, heart rate 79 beats per minute, temperature 98.9 °F (37.2 °C), respiratory rate 17 per minute, and SpO2 of 98%. There was no significant abnormality in laboratory workup, as in Table 1.

**Table 1:** Laboratory values MCV: mean corpuscular volume; MCH: mean corpuscular hemoglobin; MCHC: mean corpuscular hemoglobin concentration; RDW: red cell distribution width; BUN: blood urea nitrogen; eGFR: estimated glomerular filtration rate; CKD-EPI: Chronic Kidney Disease Epidemiology Collaboration; POC: point of care

Test	Patient Values	Reference Ranges
White blood count	7.14	4.00 - 10.00 K/uL
Red blood count	4.77	4.00 - 5.20 M/uL
Hemoglobin	15.1	12.0 - 16.0 g/dL
Hematocrit	44.4	37.0 - 47.0%
MCV	93.1	82.0 - 103.0 fL
MCH	31.7	26.0 - 34.0 pg
MCHC	34.0	30.0 - 37.0 g/dL
RDW	12.5	11.5 - 14.5%
Platelet count	329	150 - 399 K/uL
Auto neutrophil	4.16	1.84 - 7.80 K/uL
nRBC /100 WBCs	0	0 /100 WBCs
Neutrophil %	58.2	46.0 - 78.0%
Lymphocyte %	33.6	18.0 - 52.0%
Monocyte %	6.2	3.0 - 10.0%
Eosinophil %	0.6	0.0 - 6.0%
Basophil %	0.7	0.0 - 3.0%
Neutrophil	4.16	1.84 - 7.80 K/uL
Lymphocyte	2.40	0.72 - 5.20 K/uL
Monocyte	0.44	0.12 - 1.00 K/uL
Eosinophil	0.04	0.00 - 0.60 K/uL
Basophil	0.05	0.00 - 0.30 K/uL
Immature granulocyte	0.05	0.00 - 0.15 K/uL
Immature granulocyte %	0.7	0.0 - 1.5 %
Sodium	141	137 - 147 mmol/L
Potassium	4.5	3.4 - 5.3 mmol/L
Chloride	110 High	99 - 108 mmol/L
CO2 Total	23	22 - 29 mmol/L
Anion gap	8	8 - 16
BUN	9	8 - 21 mg/dL
Creatinine	0.73	0.65 - 1.00 mg/dL
BUN/Creatinine ratio	12.3	8.0 - 25.0
eGFR (CKD-EPI)	>90	>90
Lipase	23	10 - 52 U/L
POC urine pregnancy	Negative	Negative

**Figure 1:**
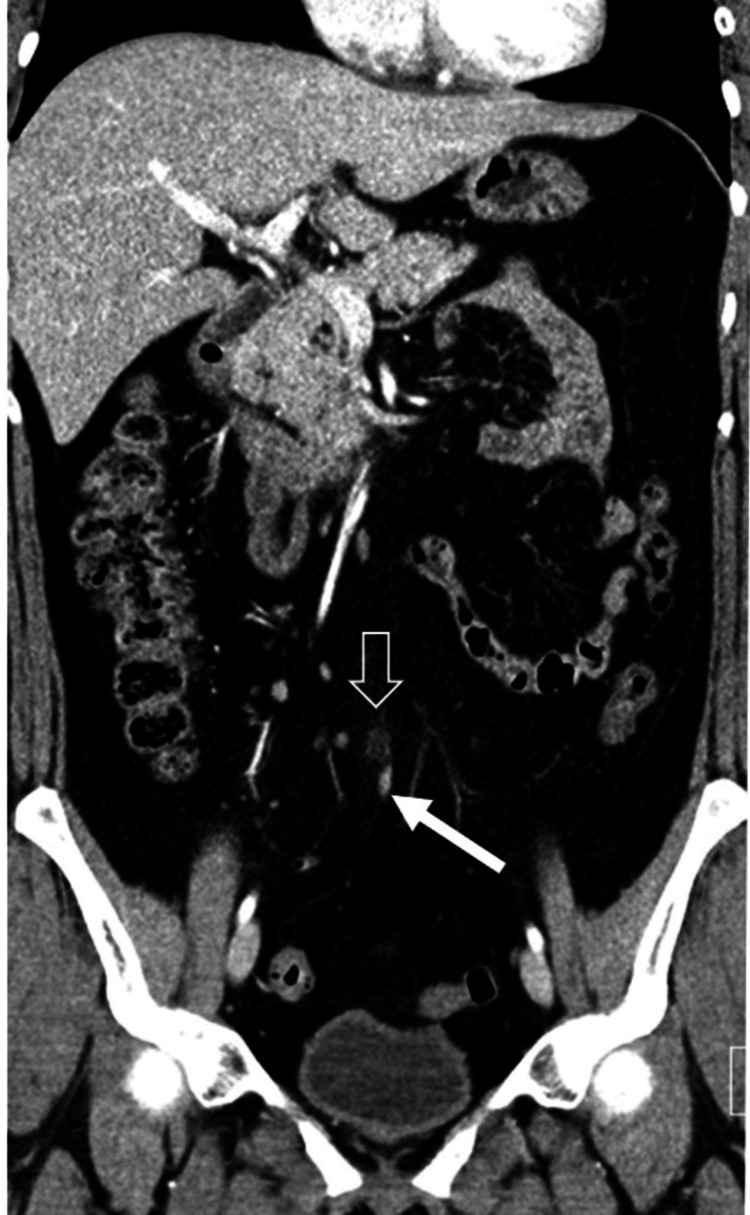
CT of the abdomen and pelvis with contrast (coronal view) showing mild focal dilation at the appendiceal tip with subtle periappendiceal fat stranding, suggesting early-stage tip appendicitis (transparent white arrow). The remainder of the appendix appears unremarkable (solid white arrow).

**Figure 2:**
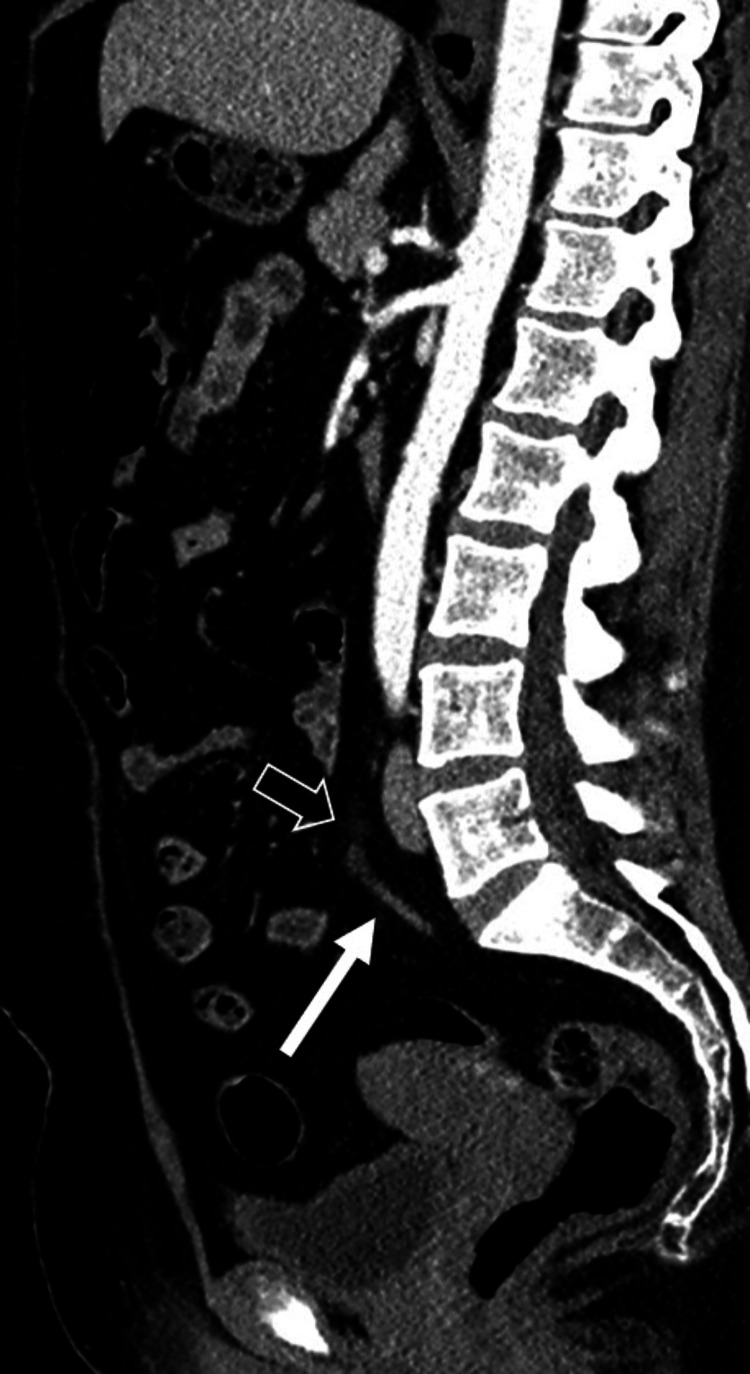
CT of the abdomen and pelvis with contrast (sagittal view) showing mild focal dilation of the appendiceal tip with subtle periappendiceal fat stranding (transparent white arrow). The rest of the appendix appears unremarkable (solid white arrow), further supporting the suspicion of early-stage tip appendicitis.

**Figure 3:**
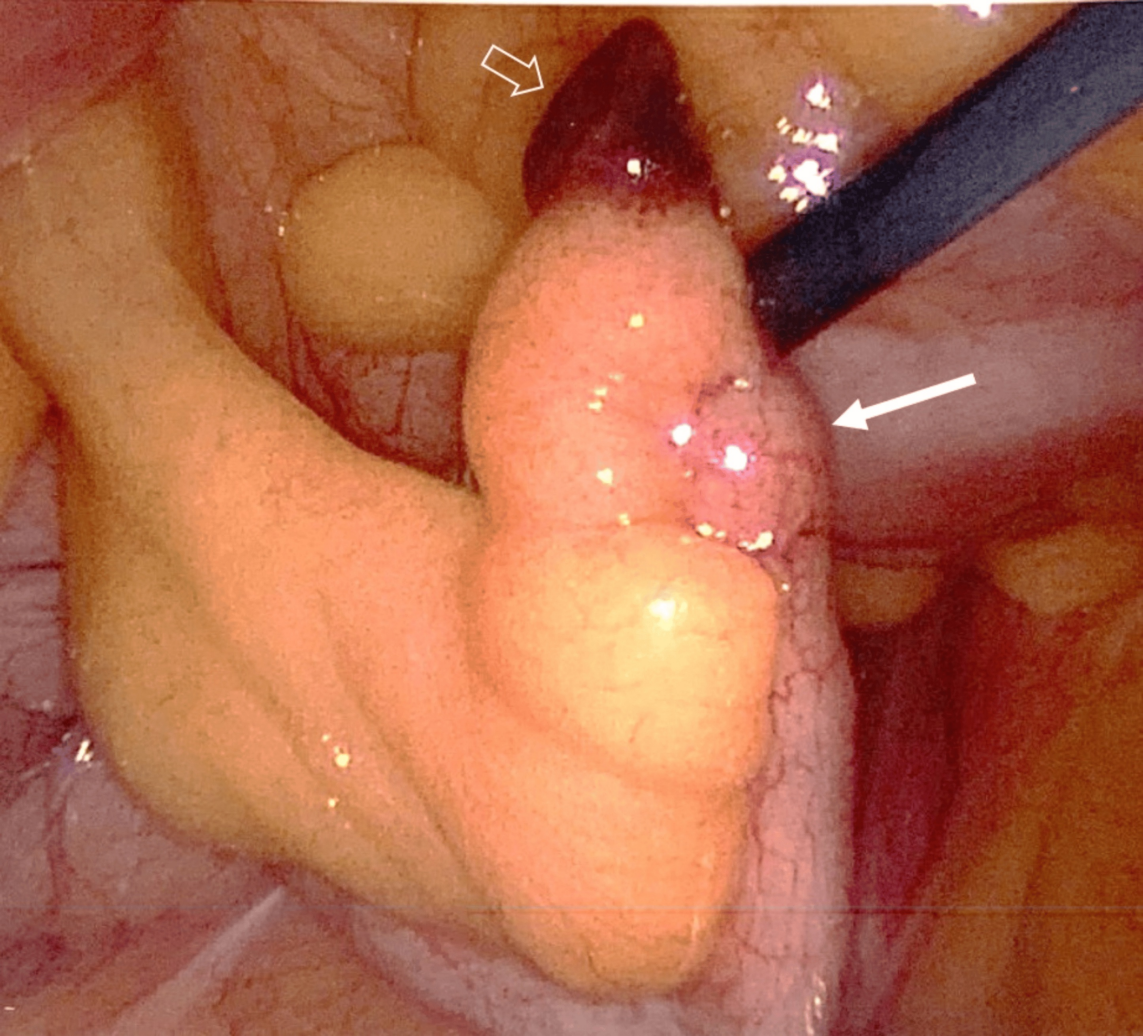
Intraoperative image of laparoscopic appendectomy, showing inflammatory changes at the tip of the appendix (transaparent white arrow). The remainder of the appendix appears normal (solid white arrow), confirming the diagnosis of tip appendicitis.

A CT scan of the abdomen and pelvis with contrast revealed mild focal dilation at the tip of the appendix, accompanied by subtle periappendiceal fat stranding, suggesting early-stage appendicitis at the tip. The remainder of the appendix appeared normal on the scan (Figure 1-2). The patient subsequently underwent a laparoscopic appendectomy, which confirmed inflammatory changes at the tip of the appendix, while the rest of the appendix appeared normal, confirming the diagnosis of tip appendicitis (Figure 3). The patient had a good postoperative outcome with complete resolution of symptoms and no complications.

## Discussion

Acute appendicitis remains a leading cause of abdominal pain, particularly in younger individuals. While the classic clinical presentation is well-established, variations such as acute tip appendicitis are much rarer and pose a diagnostic challenge [6]. This report highlights the case of a 32-year-old female patient with sharp, constant abdominal pain localized to the right lower quadrant and suprapubic region. Though her symptoms were suggestive of appendicitis, the absence of fever, nausea, and vomiting, along with minimal tenderness on physical examination, made the diagnosis less clear. As a result, CT imaging became essential in identifying the rare form of acute tip appendicitis.

CT imaging is the gold standard for diagnosing appendicitis due to its high sensitivity of 94% and specificity of 95% [5]. It plays a crucial role in detecting unusual presentations of appendicitis, including tip appendicitis. In this case, CT revealed mild dilation of the appendiceal tip along with subtle periappendiceal fat stranding, which is characteristic of early appendicitis [7]. The unremarkable appearance of the remainder of the appendix on CT helped confirm the diagnosis of acute tip appendicitis, a diagnosis that could have easily been missed without imaging. While there is no universally standardized diagnostic criterion for tip appendicitis, it is typically identified on imaging such as CT or ultrasound, which may reveal localized inflammation at the tip of the appendix, a normal-appearing base, and isolated enlargement or hyperemia of the distal portion, often with periappendiceal fat stranding or fluid confined to the tip [4,6].

Acute tip appendicitis is a relatively rare form of appendicitis, but its frequency is expected to rise as the use of CT scans becomes more widespread. Previous literature has shown that while acute tip appendicitis accounts for a small percentage of all appendicitis cases, it can still present with symptoms similar to those of typical appendicitis, including localized pain in the lower quadrants [4]. In a study by Mazeh et al., involving 18 patients with CT findings suggestive of tip appendicitis, only 39% were ultimately confirmed to have appendicitis upon clinical and/or surgical evaluation [4]. The ability to identify tip appendicitis early is essential, as the condition carries similar risks of complications such as perforation, abscess formation, and generalized peritonitis, albeit with less severe initial symptoms [8].

Timely intervention is critical to prevent the escalation of symptoms and associated morbidity. In this case, laparoscopic appendectomy confirmed the CT findings, with inflammation localized to the tip of the appendix. The laparoscopic approach is considered the standard of care for appendicitis, offering advantages such as quicker recovery, smaller incisions, and reduced postoperative pain. The patient's favorable outcome following surgery underscores the importance of prompt diagnosis and surgical intervention in even the most unusual forms of appendicitis.

Balthazar et al. demonstrated the crucial role of high-resolution CT in diagnosing acute appendicitis, with a sensitivity of 98%, specificity of 83%, and accuracy of 93% [9]. Their study highlighted CT's ability to effectively diagnose appendicitis in 64 out of 100 patients and rule it out in 31, helping to identify alternative conditions in cases where the clinical presentation was ambiguous. Furthermore, the correlation of CT findings with surgical and pathologic results confirms its reliability in both confirming appendicitis and guiding the diagnosis of other abdominal pathologies, making it an essential tool in clinical practice [10,11].

Despite the increasing use of CT, some challenges remain in diagnosing acute tip appendicitis. In many cases, it can present with less pronounced or atypical symptoms compared to more classic cases of appendicitis. This makes it crucial for clinicians to maintain a high index of suspicion, particularly when faced with right lower quadrant pain without other typical symptoms of appendicitis. A thorough differential diagnosis that includes rare forms like tip appendicitis is essential for achieving the best patient outcomes.

Numerous studies have investigated the role of CT in diagnosing appendicitis, with many emphasizing its effectiveness in identifying even rare forms like tip appendicitis. For instance, Levine et al. highlighted potential pitfalls in the CT diagnosis of appendicitis, noting that subtle findings such as mild dilation of the appendiceal tip could be easily overlooked without careful imaging interpretation [3].

Mazeh et al. reviewed the clinical implications and management of tip appendicitis, underscoring that although this condition is uncommon, its presentation often mirrors that of typical appendicitis [4]. Because tip appendicitis involves the same pathophysiological processes as more generalized appendicitis, it carries similar risks of morbidity and requires prompt intervention to avoid complications [5].

Recent studies have explored non-operative management of appendicitis, including tip appendicitis, with antibiotics alone as a potential alternative to surgery in selected cases [12]. This approach may be considered in patients with mild symptoms, low inflammatory markers, and radiological findings limited to the distal appendix. Antibiotic therapy aims to reduce inflammation and avoid surgical intervention, particularly in patients with low clinical suspicion for perforation or complications. However, careful clinical judgment and close follow-up are essential, as some cases may still progress and require surgical management [12].

Overall, while tip appendicitis is a rare condition, it is becoming increasingly identifiable due to the widespread use of advanced imaging techniques like CT. As this case demonstrates, early detection and surgical intervention are critical to preventing complications. Further research and continued clinical awareness are needed to refine diagnostic criteria and management protocols for this uncommon yet important variant of appendicitis.

## Conclusions

Acute tip appendicitis, though a rare variant, presents a diagnostic challenge due to its subtle and often nonspecific clinical presentation. This case underscores the importance of including tip appendicitis in the differential diagnosis of right lower quadrant pain, especially as the use of CT imaging becomes increasingly prevalent. CT is an invaluable tool in detecting even subtle cases of tip appendicitis. While surgical intervention, particularly laparoscopic appendectomy, remains the standard of care when clinical suspicion is high, emerging evidence suggests that conservative antibiotic therapy may also be appropriate in selected, uncomplicated cases. Early identification through imaging is crucial to initiate appropriate management and prevent complications such as perforation or abscess formation. Despite its rarity, recognizing tip appendicitis is essential, as it carries similar risks of morbidity as typical appendicitis and requires prompt, informed intervention to ensure optimal outcomes.
